# Hypercrosslinked porous polymers hybridized with graphene oxide for water treatment: dye adsorption and degradation†

**DOI:** 10.1039/c8ra01620h

**Published:** 2018-04-10

**Authors:** Yipeng Huang, Guihua Ruan, Yuji Ruan, Wenjuan Zhang, Xianxian Li, Fuyou Du, Cunjie Hu, Jianping Li

**Affiliations:** Colleges and Universities Key Laboratory of Food Safety and Detection, College of Chemistry and Bioengineering, Guilin University of Technology Guilin Guangxi 541004 China guihuaruan@hotmail.com; Collaborative Innovation Center for Water Pollution Control and Water Safety in Karst Area Guilin Guangxi 541004 China; School of Biomedical Engineering, Southern Medical University Guangzhou Guangdong 510515 China

## Abstract

Hypercrosslinked porous polymer hybridized graphene oxide with polymeric high internal phase emulsions (polyHIPEs/GO) were designed as versatile composites for water treatment. Morphologies, chemical composition and thermal stability of the composites were characterized by SEM, FTIR, XPS, XRD and TGA. Tunable adsorption properties and enhanced visible-light photocatalysis towards organic dyes were achieved by the manipulation of functional groups and the inclusion of Ag_3_PO_4_, respectively. The adsorption capacity of polyHIPEs/GO towards cationic methyl blue (MB) and rhodamine B (RB) is 1250.3 and 1054.1 μg g^−1^, respectively. Aminated polyHIPEs/GO (polyHIPEs_(NH_2_)_/GO) possesses an adsorption capacity of 1967.3 μg g^−1^ to anionic eosin Y (EY). The tandem columns of polyHIPEs_(NH_2_)_/GO and polyHIPEs/GO can successively and selectively remove the cationic and anionic dyes in a mixed dye solution. Furthermore, enhanced photodegradation ability was obtained after GO reduction and Ag_3_PO_4_ addition on polyHIPEs_(NH_2_)_/GO. Results show that 3.5 × 10^−5^ M of MB, RB and EY can be completely photodegraded by 20 mg of the novel photocatalyst within 20, 40 and 35 min, respectively. This work demonstrates that polyHIPEs/GO exhibits tunable properties for multiply progressive applications in water treatment and catalysis.

## Abstract 2

We report the synthesis of graphene oxide hybridized polymeric high internal phase emulsions and their applications in adsorption and photocatalysis.

## Graphical abstract

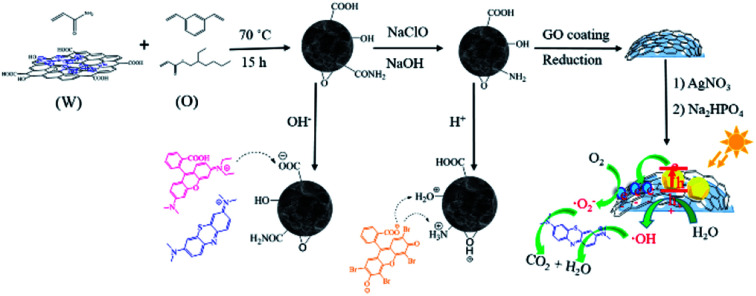

## 1. Introduction

Graphene has attracted significant attention in many disciplines due to its impressive surface area, extreme mechanical strength, high thermal and high electron mobility.^1,2^ Assembling 2D graphene derivatives into macroscopic 3D structures is an essential step to expand their practical applications in oil or contaminant treatment,^3–5^ catalysis,^6,7^ sensing^8–10^ and energy storage.^3,11^ However, many 3D macroporous graphene materials collapse easily, and the assembly of a robust structure is still a challenge.^2^ In recent years, numerous attempts have been made to incorporate graphene oxide (GO) and/or reduced graphene oxide (RGO) into functional polymers for mechanical enhancement. Besides, the resultant graphene–polymer hybrids not only retain some physiochemical properties of the individual components, but also are endowed with some novel characters that are different from those individual components. On account of this, GO–PVP, GO–PVA, GO–PEO, *etc.* have been reported.^12–15^ However, the preparation of these GO–polymer 3D networks relies on high quantities of GO matrix, which results in high fabricating costs.

As an effective method to fabricate macroporous polymers, high internal phase emulsions (HIPEs) have been intensively exploited.^16^ Polymers obtained from HIPE templates which are known as polyHIPEs are often endowed with high porosity, good permeability, and functional group tenability.^17,18^ Fabricating GO hybridized polyHIPEs (polyHIPEs/GO) is seemingly a great option to improve the structural stability of macropores and to impart many organic groups to the resultant polymers. In addition, the preparation of polyHIPEs/GO can greatly reduce the GO quantity used, which is cost-effective. But surprisingly, polyHIPEs/GO has rarely been exploited in the past years.

In this work, we firstly report the synthetic and surface manipulation strategies of polyHIPEs/GO. Then, we study the morphologies, chemical composition and thermal stability of the polymers using scanning electron microscope (SEM), Fourier transform infrared spectroscopy (FTIR), X-ray photoelectron spectroscopy (XPS), X-ray diffraction (XRD) and thermo gravimetric analysis (TGA). Furthermore, we show that the as-prepared polyHIPEs/GO is useful in dye adsorption and photodegradation.

## 2. Experimental

### 2.1 Materials

Methyl blue (MB, AR), rhodamine B (RB, AR), eosin Y (EY, AR), graphite powders (99.95%), 2-ethylhexyl acrylate (EHA, 99%), acrylamide (AAm, 99%), divinylbenzene (DVB, 98%), sorbitan monooleate (Span 80), polyvinylpyrrolidone (PVP, K-30), azo-bisisobutyronitrile (AIBN), and ascorbic acid (AA) were purchased from Aladdin Chemistry Co. Ltd (Shanghai, China). Sulfuric acid (H_2_SO_4_, 98%), hydrogen peroxide (H_2_O_2_, 30%), hydrochloric acid (HCl, 37%), ethanol (AR), sodium nitrate (NaNO_3_, AR), potassium permanganate (KMnO_4_, AR), sodium hydroxide (NaOH, AR), sodium hypochlorite solution (NaClO, 5.5% effective chlorine), silver nitrate (AgNO_3_, AR), sodium phosphate dibasic dodecahydrate (Na_2_HPO_4_˙ 12H_2_O, AR) were supplied by Chengdu XiYa Chemical Technology Co., Ltd. (Sichuan, China). Deionized (DI) water was used throughout.

### 2.2 GO preparation and modification

Graphite oxides were prepared according to the previous modified Hummer's method.^19^ Aqueous GO suspension (5.0 mg mL^−1^) was obtained by ultrasonicating the graphite oxide dispersion for 1 h. Then, 667 mg PVP was added into 20 mL of GO suspension, followed by magnetic stirring for 12 h at room temperature,^20^ then centrifuged at 15 000 rpm for 20 min to remove the unbound PVP. The collected slurry was redispersed in DI water with a final volume of 20 mL.

### 2.3 Synthesis of polyHIPEs/GO

An oil phase consisting of 400 μL of EHA, 300 μL of DVB, 15 vol% (with respect to the whole volume of oil phase) of Span 80, and 1.5 wt% (with respect to the total mass of EHA and DVB) of initiator AIBN was added to a 3.2 mL of aqueous phase containing 100 mg of AAm and 2.0 mL of PVP–GO in a 10 mL polypropylene centrifuge tube. The mixtures were emulsified with an MS-3B homogenizer (IKA, Germany) at 3000 rpm for 5 min to form HIPEs (80% internal phase). HIPEs with identical internal volume ratio but different AAm, DVB, and PVP–GO quantity were prepared as described in Table S1. The HIPEs were polymerized at 70 °C for 15 h to yield polyHIPEs/GO porous monoliths. After cooled to room temperature, the unreacted components were eliminated with ethanol and DI water, then the polymers were lyophilized at −50 °C and <20 Pa for 24 h.

### 2.4 Amination and Ag_3_PO_4_ decoration on polyHIPEs/GO

Hoffman reaction^21^ was used for the transformation of amide groups to primary amine groups. Briefly, 1.0 g polyHIPEs/GO monolith was added to 10 mL ice water. Subsequently, 2.0 mL of NaOH solution (1 M) and 2.0 mL of NaClO solution (5.5% effective chlorine) were added. The reaction was carried out at 0 °C for 6.5 h, then heated at 70 °C for 1.5 h with gently shaking. After the reaction, the excess NaOH and NaClO were eliminated with DI water, and the products were lyophilized. The obtained aminated polyHIPEs/GO is denoted as to polyHIPEs_(NH_2_)_/GO.

The monolithic polyHIPEs_(NH_2_)_/GO was crushed into powders and the powders passing through the sieve (100 meshes per cm^2^) were collected. The collected powders (50 mg) were coated with additional GO (5.0 mg) *via* self-assembly in 10 mL of water, then the GO was reduced by AA (50 mg) in a 25 mL Teflon-lined stainless steel autoclave at 95 °C for 3 h.^22^ After the reaction, the autoclave was left to cool naturally to room temperature, then washed with DI water and collected by centrifugation. The obtained polyHIPEs_(NH_2_)_/RGO was dispersed in 20 mL of water, and AgNO_3_ aqueous solution (2.0 mL, 0.6 M) was added dropwise with magnetic stirring. After the addition, the suspension was kept stirring for further 12 h to ensure the adsorption of Ag^+^ on the surface of polyHIPEs_(NH_2_)_/RGO. Then Na_2_HPO_4_ aqueous solution (2.0 mL, 0.2 M) was added dropwise to the mixture, and the mixture was kept stirring for 30 min.^23^ PolyHIPEs_(NH_2_)_/RGO/Ag_3_PO_4_ composites were obtained by collecting and freeze drying the centrifugal precipitates.

### 2.5 Characterizations

Atomic force microscope (AFM) images of PVP–GO were obtained using a Multimode 8 in the tapping mode. The PVP–GO sample was dispersed in water and spin coating onto freshly cleaved mica substrates before the test. FTIR spectra were recorded in KBr pellets using an IS10 FTIR spectrometer (Thermo Fisher Scientific Co., USA). Surface morphologies of the samples were observed by a SU5000 field emission SEM (Hitachi Ltd, Japan). Powder XRD spectra were recorded using a PANalytical X′ Pert^3^ Powder diffractometer with Cu Kα radiation at 40 kV and 40 mA, and a scanning rate of 5° (2*θ*)/min from 10° to 80°. TGA was carried out using TA Instruments (SDT Q 600) at a heating rate of 10 °C min^−1^ from 25 °C to 600 °C in nitrogen atmosphere. XPS spectra were obtained with an Axis Ultra DLD (Kratos Ltd, U.K.) paired with a monochromatic Al Kα X-ray source (1486.6 eV).

### 2.6 Dye adsorption and desorption

Two cationic dyes (MB and RB) and one anionic dye (EY) were employed to investigate the adsorption behaviors of the polyHIPEs/GO and polyHIPEs_(NH_2_)_/GO. Typically, the monolithic sorbent (0.1 g) was added into aqueous dye solution (20 mL of 10 μg mL^−1^), followed by gently shaking at room temperature. At predetermined time intervals, the dye concentration remaining in the solution was measured using a TU-1901 UV-Vis spectrophotometer at the maximum absorbance of each dye (MB: 664 nm; RB: 557 nm; EY: 515 nm). The adsorption amount at time *t*, *q*_*t*_ (μg g^−1^), was calculated using the following equation:*q*_*t*_ = *V*(*C*_0_ − *C*_*t*_)/*m*where *V* is the volume of dye solution (mL); *C*_0_ and *C*_*t*_ (μg mL^−1^) are the dye concentration initially and at time *t*, respectively; and *m* stands for the mass of the sorbent (g).

**Fig. 1:**
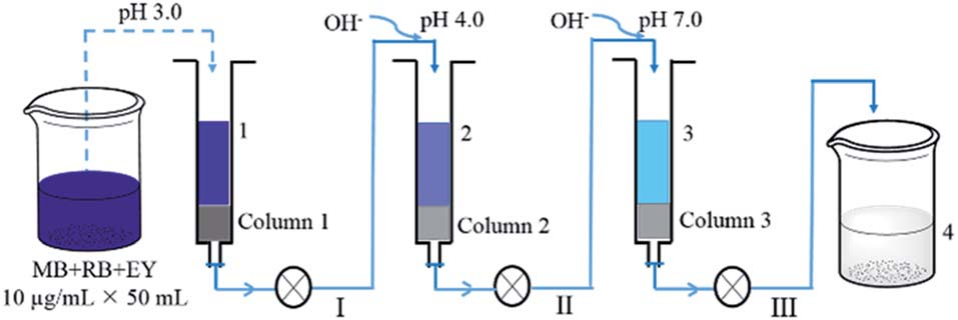
Schematic illustration of the adsorption of mixed dye solution by polyHIPEs_(NH_2_)_/GO (column 1) and polyHIPEs/GO (column 2 and 3) tandem columns.

A mixed dye solution (50 mL) containing 10 μg mL^−1^ of MB, RB and EY was successively passed through a polyHIPEs_(NH_2_)_/GO column and two polyHIPEs/GO columns under different pH values (Fig. 1). The columns used in all processes were 0.2 g in weight and ∼4 mm in length, and the filtration rate is controlled at 0.1 mL min^−1^. The pH of the solution was adjusted using 0.1 MHCl and NaOH. UV-Vis spectra of the solution at different filtration stages were all recorded.

### 2.7 Photocatalytic study

A 350 W Xe lamp (XPA-4 Photoreactor) equipped with a cutoff filter (*λ* > 420 nm) was used as irradiation source. Dye solutions (20 mL, 3.5 × 10^−5^ M) containing 20 mg of photocatalysts were put in a cylindrical glass beaker and stirred in the dark to reach absorption–desorption equilibrium. Next, Xe lamp was turned on to start the photocatalytic reaction. At regular time intervals, Xe lamp was turned off and 4 mL of the solution was taken out and centrifuged to separate the photocatalyst. Then UV-Vis spectra of the supernatant were recorded. After that, the solution was pour back in the beaker. The photocatalytic reaction and supernatant analysis processes were repeated until the dyes were degraded completely. The degradation efficiency was evaluated by *C*_*t*_/*C*_0_. Here, *C*_*t*_ and *C*_0_ are the concentration (M) of dyes at time *t* (min) and initially.

## 3. Results and discussion

### 3.1 Characterization of polymers and composites

**Fig. 2:**
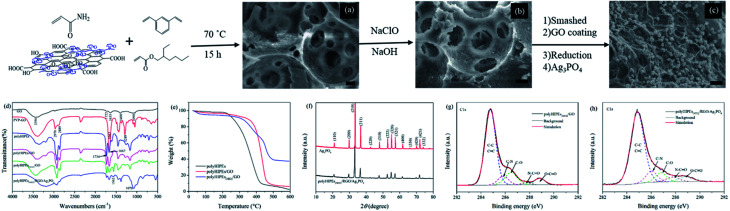
Synthesis processes and characterization. SEM images of polyHIPEs/GO (a), polyHIPEs_(NH_2_)_/GO (b), and polyHIPEs_(NH_2_)_/RGO/Ag_3_PO_4_ (c). Scale bars all are 10 μm. FTIR spectra of the prepared materials (d), TGA curves for polyHIPE, polyHIPEs/GO and polyHIPEs_(NH_2_)_/GO (e), XRD patterns for Ag_3_PO_4_ and polyHIPEs_(NH_2_)_/RGO/Ag_3_PO_4_ (f), and high resolution C1s XPS spectra of polyHIPEs/GO (g) and polyHIPEs_(NH_2_)_/RGO/Ag_3_PO_4_ (h). In these polyHIPEs/GO, polyHIPEs_(NH_2_)_/GO and polyHIPEs_(NH_2_)_/RGO/Ag_3_PO_4_ samples, the GO, RGO and Ag_3_PO_4_ contents are 1.35 wt%, 4.6 wt% and 49.7 wt%, respectively.

The dispersibility and sheet thickness of PVP–GO were collected using atomic force microscopy. As shown in Fig. S1,† PVP–GO sheets remain good dispersibility in water, and the thickness is less than 1 nm. The sheet edge tends to curl up, causing the significant increase in vertical thickness. Morphologies of the HIPE-based polymers and Ag_3_PO_4_-based composites were observed by SEM. Fig. 2a and b reveal the open-cell porous structure of polyHIPEs/GO and polyHIPEs_(NH_2_)_/GO. Spherical voids with tens of microns in diameter are derived from the emulsion droplets. Many circular windows existing in the void surface interconnect the adjacent voids. Besides, a myriad of nanoscale to submicroscale grooves are also found in the wall (Fig. S2†). Because the relatively low PVP–GO content and the non-transparency of the polyHIPEs matrix, it is unfeasible to directly observe the distribution of PVP–GO sheets under SEM or TEM, unless the PVP–GO sheets are aggregated on the surface. According to Fig. 2a and b, no aggregated PVP–GO sheets can be seen, we can presume that the amphiphilicity of PVP–GO is uniformly located at the surface between oil phase and aqueous phase of an emulsion. TGA results (Fig. 2e) reveal that the incorporation of GO can apparently enhance the thermal stability of the polymers. The thermal degradation of polyHIPEs and polyHIPEs/GO happens mainly in the range of 230–430 °C and 350–480 °C, respectively. This in turn demonstrates our previous presumption, because GO can act as an excellent barrier to protect the inner polymer matrix from thermal degradation before 350 °C only when GO is uniformly located at the surface.^24^ The amination further increases the degradation temperature (37% of the weight for polyHIPEs_(NH_2_)_/GO is remained even at 600 °C), revealing the higher thermal stability of amines to amides. In Fig. 2c, as the RGO content is increased (compared with the GO content in Fig. 2a and b), the RGO layer is observable. Fig. 2c clearly shows the hierarchical structure of polyHIPEs_(NH_2_)_/RGO/Ag_3_PO_4_. The RGO layer avoids the direct contact of polyHIPEs_(NH_2_)_ and Ag_3_PO_4_. The particle size of Ag_3_PO_4_ in polyHIPEs_(NH_2_)_/RGO/Ag_3_PO_4_ hybrids has a distinct decrease compared with that of bared Ag_3_PO_4_ (Fig. S3†). This phenomenon is consistent with the precious report which revealed that GO has an obvious effect on the size of Ag_3_PO_4_ particles.^23^ The XRD pattern (Fig. 2f) for polyHIPEs_(NH_2_)_/RGO/Ag_3_PO_4_ clearly shows all characteristic diffraction peaks that could be readily indexed to the body-centered cubic structure of Ag_3_PO_4_ (JCPDS no. 06-0505), demonstrating the same structure of Ag_3_PO_4_ in these two samples despite their different particle sizes.

In Fig. 2d, the FTIR spectrum of GO shows absorption bands at 1722 cm^−1^ (*ν*_OC=O_), 1400 cm^−1^ (*ν*_C–OH_), and 1090 cm^−1^ (*ν*_C–O–C_), depicting the existence of the carboxyl, hydroxyl and epoxy groups.^25^ Absorption peaks at 2970 cm^−1^ (*ν*_C–H_), 1663 cm^−1^ (*ν*_NC=O_) and 1289 cm^−1^ (*ν*_N–C_)^26^ in the FTIR spectrum of PVP–GO confirms the PVP chains were successfully modified onto the GO sheets. According to the change in relative peak intensity at 1734 cm^−1^ (*ν*_OC=O_) and 1667 cm^−1^ (*ν*_NC=O_) in FTIR spectra of polyHIPEs and polyHIPEs/GO, we can presume the presence of PVP–GO in the polymer matrix. In the FTIR spectrum of polyHIPEs_(NH_2_)_/GO, the decreased absorption at 1667 cm^−1^ (*ν*_NC=O_) and the appeared absorption at 1562 cm^−1^ (*δ*_N–H_) proves that amides have translated to amines.^21^ The absorption peak corresponded to PO_4_^3−^ (1076 cm^−1^) in the FTIR spectrum of polyHIPEs_(NH_2_)_/RGO/Ag_3_PO_4_ demonstrates the presence of Ag_3_PO_4_ in the hybrids.^27^ To prove the GO has been reduced in polyHIPEs_(NH_2_)_/RGO/Ag_3_PO_4_, high resolution C1s XPS spectra of polyHIPEs_(NH_2_)_/GO and polyHIPEs_(NH_2_)_/RGO/Ag_3_PO_4_ samples were collected (Fig. 2g and h). Peak intensity corresponding to carbons singly bonded to epoxy/hydroxyls (C–O, 286.5 eV) and carbons in carboxyl/ester groups (O–C=O, 288.9 eV)^22^ in the C1s XPS spectrum of polyHIPEs_(NH_2_)_/RGO/Ag_3_PO_4_ is decreased compared with that in polyHIPEs_(NH_2_)_/GO, confirming the reduction of GO. The remaining intensity at 286.5 and 288.9 eV is ascribed to the C–O and O–C=O from the polymer matrix that cannot be reduced by ascorbic acid. The reduction of oxygen-containing groups on GO sheets forms C=C, leading to a slightly broadening to higher binding energy in the peak associated with C–C and C=C.

### 3.2 Dye adsorption

**Fig. 3:**
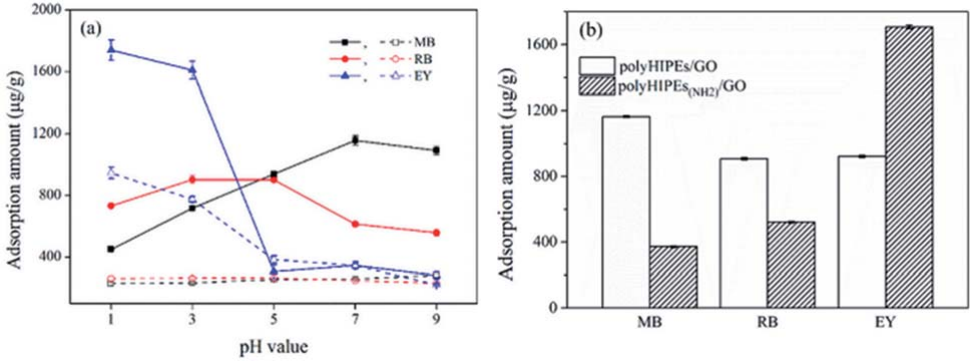
Investigation of pH on the adsorption of dyes. (a) Effect of pH on the adsorption capacity of polyHIPEs (dashed line) and polyHIPEs/GO (solid line) to MB and RB, and polyHIPEs_(NH_2_)_ (dashed line) and polyHIPEs_(NH_2_)_/GO (solid line) to EY, (b) adsorption capacity of polyHIPEs/GO and polyHIPEs_(NH_2_)_/GO under optimized pH. The GO content in polyHIPEs/GO and polyHIPEs_(NH_2_)_/GO is 1.35 wt%.

Dye removal behaviors of monolithic polyHIPEs/GO and polyHIPEs_(NH_2_)_/GO were investigated using two cationic dyes (MB and RB) and an anionic dye (EY) as models. Since the adsorption of dyes on these sorbents is expected to have great connections with surface charge, effect of pH on the adsorption capacity was firstly investigated. As shown in Fig. 3a, the adsorption of MB and RB on polyHIPEs (without any GO) is rather weak and varies little as the pH is increased. Differently, the adsorption of EY on polyHIPEs_(NH_2_)_ decreases when the pH is increased from 1.0 to 5.0, then remains a constant. After GO is combined with polyHIPEs or polyHIPEs_(NH_2_)_, the adsorption capacity to these dyes enhances significantly. The adsorption of cationic MB on polyHIPEs/GO enhances with the increase in pH value, and the maximum adsorption amount is obtained when the pH ≥ 7.0, because the deprotonation of carboxyl and hydroxyl groups trigger the electrostatic interactions (Fig. S4†). Differently, the maximum adsorption of RB appears at the pH range of 3.0–5.0. This is because the highest deprotonation of carboxyl groups in RB molecules at high pH range inversely leads to the weak positive charge of the whole molecule, weakening the electrostatic interactions. Strongest electrostatic interaction between RB molecule and polyHIPEs/GO is achieved under weakly acidic condition due to carboxyl groups are partially deprotonated. When we use polyHIPEs_(NH_2_)_/GO to absorb the anionic EY, the highest adsorption amount is achieved when the pH ≤ 3.0, where amine, hydroxyl and epoxy on the sorbent are protonated, leading to a strong electrostatic interaction with the anionic EY. Under the optimized pH, the maximum adsorption amount of polyHIPEs_(NH_2_)_/GO to MB and RB is significantly lower than that of polyHIPEs/GO, while the maximum adsorption amount of polyHIPEs_(NH_2_)_/GO to EY is much higher than that of polyHIPEs/GO (Fig. 3b), well demonstrating the reasonability of employing polyHIPEs/GO and polyHIPEs_(NH_2_)_/GO for the adsorption of cationic dyes and anionic dyes, respectively.

**Fig. 4:**
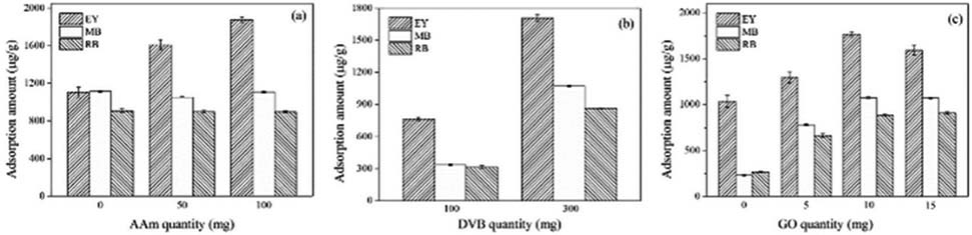
Effects of the quantity of AAm (a), DVB (b) and GO (c) on the adsorption capacity of polyHIPEs/GO to MB and RB, and polyHIPEs_(NH_2_)_/GO to EY.

To understand the adsorption mechanism in depth, the quantity of AAm, DVB and GO were investigate. It was found that the adsorption capacity of polyHIPEs_(NH_2_)_/GO to EY heightens with the ascendent quantity of AAm, however, the increased AAm quantity has less effects on the adsorption capacity of polyHIPEs/GO to MB and RB (Fig. 4a). This prove that the amide groups have little effects on the adsorption of cationic dyes. When amide groups are transformed to amine groups, the protonated amine groups contribute to the electrostatic attraction with the anionic EY. The increase in DVB quantity ends with higher adsorption capacity of both polyHIPEs/GO and polyHIPEs_(NH_2_)_/GO (Fig. 4b), revealing the π–π interaction adsorption mechanism. Increasing in GO quantity can enhance both the electrostatic interaction and π–π interaction, which is supported by the result that the adsorption amount of MB, RB, and EY all ascends when the GO quantity rises from 0 to 10 mg (Fig. 4c). Nonetheless, further increase in GO quantity cannot obtain higher adsorption capacity probably due to the aggregation of GO and some of the GO sheets are unable to show their functions.

The dye adsorption on polyHIPEs/GO and polyHIPEs_(NH_2_)_/GO follows the pseudo-second-order kinetic model (Fig. S5†). The saturated adsorption capacity (*q*_*e*_) of polyHIPEs/GO to MB and RB, polyHIPEs_(NH_2_)_/GO to EY are calculated to be 1250.3, 1054.1, 1967.3 μg g^−1^, respectively. The adsorption rate constants (*k*) to MB, RB and EY are 2.30 × 10^−4^, 2.38 × 10^−4^ and 1.49 × 10^−4^ (Table S2). In addition, the polymer sorbents adsorbed with dyes are ease of releasing the dyes in ethanol (desorption efficiencies > 87%), and thus possess good cycling performance (Fig. S6† and S7†). As pointed in Table S3, polyHIPEs/GO and polyHIPEs_(NH_2_)_/GO exhibit superiorities including the broad-spectrum adsorption ability, simple synthesis procedure, low-cost of the monolithic sorbent and good recycling performance when compared with the powder-like, aerogel, or hydrogel sorbents. The relatively low adsorption capacity of the as-prepared sorbents probably because the higher bulk density compared with the light weight aerogel and GO rich sorbents. When the adsorption capacity is evaluated by the dye quantity adsorbed in per gram sorbent, the light weight sorbent possessing a much larger volume benefits a higher uptake of dyes. To address the low adsorption capacity, continuous research will be carried out in the future.

**Fig. 5:**
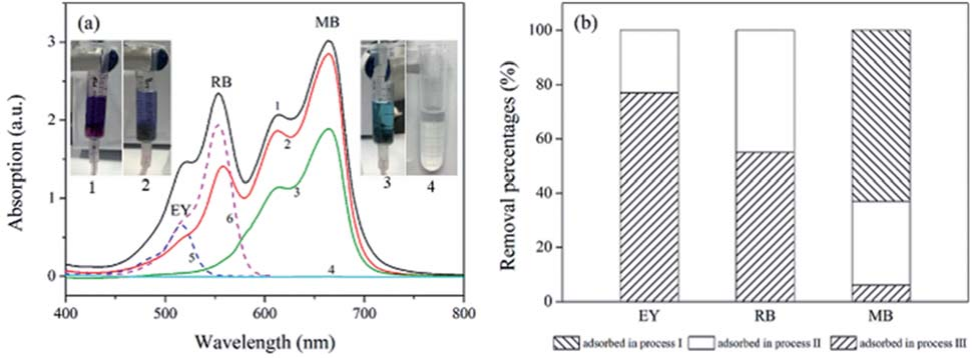
The filtration of dyes using polyHIPE_(NH_2_)_/GO and polyHIPEs/GO columns. (a) UV-Vis spectra of the dye solution at different adsorption processes. Spectra 1–4 correspond to the original mixed dye solutions (50 mL × 10 μg mL^−1^ of MB, RB, and EY), solution passed through polyHIPE_(NH_2_)_/GO column at pH 3.0, solution passed through polyHIPEs/GO column at pH 4.0, solution passed through polyHIPEs/GO column at pH 7.0, spectra 5 and 6 are solo EY and RB, respectively. (b) Adsorption percentages of EY, RB and MB in processes I–III illustrated in Fig. 1. The GO content in polyHIPEs/GO and polyHIPEs_(NH_2_)_/GO is 1.35 wt%.

The removal of dyes from water were further studied using the polyHIPEs/GO and polyHIPEs_(NH_2_)_/GO as column packing. As illustrated in Fig. 1, a mixed solution containing MB, RB and EY was successively passed through the polyHIPEs_(NH_2_)_/GO column at pH 3.0, polyHIPEs/GO column at pH 4.0, and polyHIPEs/GO column at pH 7.0. Fig. 5 showed that 77% of EY, 55% of RB and 5% of MB were filtered by polyHIPEs_(NH_2_)_/GO column at pH 3.0. Subsequently, the remaining 23% of EY, 45% of RB and 31% MB were further filtered after passing the polyHIPEs/GO column at pH 4.0. Finally, the rest of 63% of MB were eliminated by polyHIPEs/GO column at pH 7.0. This result suggests that various dyes in the polluted water can be selective filtered *via* the tandem use of polyHIPEs_(NH_2_)_/GO and polyHIPEs/GO columns. Moreover, the polluted solution can recover to neutral after such treatment.

### 3.3 Photocatalytic performance of polyHIPEs_(NH_2_)_/RGO/Ag_3_PO_4_

**Fig. 6:**
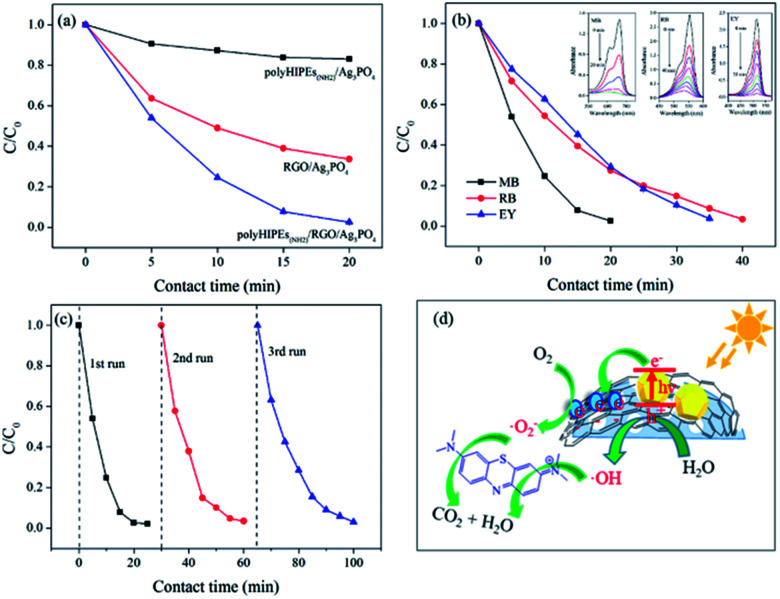
Photocatalytic performance of polyHIPEs_(NH_2_)_/RGO/Ag_3_PO_4_. (a) Photodegradation of MB over polyHIPEs_(NH_2_)_/Ag_3_PO_4_, RGO/Ag_3_PO_4_ and polyHIPEs_(NH_2_)_/RGO/Ag_3_PO_4_. (b) Photodegradation of MB, RB and EY over polyHIPEs_(NH_2_)_/RGO/Ag_3_PO_4_, and adsorption spectra of MB, RB and EY over polyHIPEs_(NH_2_)_/RGO/Ag_3_PO_4_ in inset. (c) Cycling performance for photodegradation of MB over polyHIPEs_(NH_2_)_/RGO/Ag_3_PO_4_ under visible light irradiation. (d) Schematic illustration of the photocatalytic mechanism of polyHIPEs_(NH_2_)_/RGO/Ag_3_PO_4_. (Photocatalysts: 20 mg, dye solutions: 20 mL with a concentration of 3.5 × 10^−5^ M).

Using polyHIPEs_(NH_2_)_/GO, we successfully prepared polyHIPEs_(NH_2_)_/RGO/Ag_3_PO_4_ composites and studied their photocatalytic performance. To our delight, polyHIPEs_(NH_2_)_/RGO/Ag_3_PO_4_ exhibits significantly enhanced visible-light photocatalytic activity for MB degradation than that of polyHIPEs_(NH_2_)_/Ag_3_PO_4_ and RGO/Ag_3_PO_4_ (Fig. 6a). Further study proved that the hierarchical polyHIPEs_(NH_2_)_/RGO/Ag_3_PO_4_ possesses good photodegradation ability to other dyes including RB and EY (Fig. 6b). Photodegradation efficiency of MB over polyHIPEs_(NH_2_)_/RGO/Ag_3_PO_4_ decreases slightly after three or more cycles (Fig. 6c), revealing the photocatalytic stability of polyHIPEs_(NH_2_)_/RGO/Ag_3_PO_4_. Moreover, our polyHIPEs_(NH_2_)_/RGO/Ag_3_PO_4_ (49.7 wt% of Ag_3_PO_4_) shows comparable or even higher photocatalytic efficiency to other well-known photocatalysts (mostly contain >90 wt% semiconductors), such as RGO/BiVO_4_,^28^ RGO/CdS,^29^ RGO/TiO_2_,^30,31^ GO/Ag_3_PO_4_,^27,32^ and RGO/Ag_3_PO_4_ (ref. 23) (Table S4). In this status, our polyHIPEs_(NH_2_)_/RGO/Ag_3_PO_4_ not only greatly reduces the synthesis cost, but maintains high photocatalytic activity.

It is known that the bandgap of Ag_3_PO_4_ semiconductor is 2.45 eV (conduction band, CB: +0.45 eV; valence band, VB: +2.9 eV)^33^ The possible photodegradation processes are illustrated in Fig. 6d: (1) under visible-light irradiation, the electrons at the VB of Ag_3_PO_4_ are excited to the CB, leading to the separation of electron–hole pairs. (2) Photogenerated electrons at the surface of Ag_3_PO_4_ are rapidly transferred by RGO, preventing the recombination of the electron–hole pairs and accelerating the formation of a steady flow of electron–hole pairs. (3) Once the photogenerated electrons are captured by O_2_ molecules, superoxide radicals (O_2_^−^˙) are produced. At the same time, hydroxyl radicals (·OH) generates from the reaction of H_2_O and the active holes.^23,34^ (4) Organic dyes are attracted to the surface of polyHIPEs_(NH_2_)_/RGO/Ag_3_PO_4_ due to the good adsorption behavior of the polymer, which accelerates the reaction of dye molecules and radicals. (5) By continuously working in the aforementioned manners, dye molecules are finally degraded into CO_2_, H_2_O and other small molecules.^30,35^

## 4. Conclusions

We have successfully prepared a versatile polyHIPEs/GO porous polymer with tunable properties for multiply progressive applications. This presented polyHIPEs/GO shows good adsorption performance to cationic dyes. After simply tuning the surface groups, the resultant polyHIPEs_(NH_2_)_/GO exhibits improved adsorption properties to anionic dyes. The adsorption mechanisms, adsorption kinetics, and cycling performance of these two sorbents have been systematically investigated and discussed. Furthermore, we reported a novel polyHIPEs_(NH_2_)_/RGO/Ag_3_PO_4_ with enhanced visible-light photocatalytic activity by RGO coating and Ag_3_PO_4_ decoration on the surface of polyHIPEs_(NH_2_)_/GO. Our work suggests that the new polyHIPEs/GO porous polymer can be used as sorbents, filters, and photocatalysts.

## Conflicts of interest

There are no conflicts to declare.

## Supplementary Material

RA-008-C8RA01620H-s001
