# Supplementary material for: Hypercrosslinked porous polymers hybridized with graphene oxide for water treatment: dye adsorption and degradation
Source: RSC Adv. 2018 Apr 10;8(24):13417–22. doi: 10.1039/c8ra01620h (PMC9079756; doi:10.1039/c8ra01620h)
Supplement: RA-008-C8RA01620H-s001 [file RA-008-C8RA01620H-s001.pdf]

Electronic Supplementary Material (ESI) for RSC Advances.
This journal is © The Royal Society of Chemistry 2018

Supplementary Information

for

Hypercrosslinked porous polymers hybridized with graphene oxide for water treatment: dye adsorption and degradation

Yipeng Huang[a,1] , Guihua Ruan[a,b,*], Yuji Ruan[c,1], Wenjuan Zhang[a], Xianxian Li[a], Fuyou Du[a,b], Cunjie Hu[a], Jianping Li[a,b]

a. Colleges and Universities Key Laboratory of Food Safety and Detection, College of Chemistry and Bioengineering, Guilin University of Technology, Guilin, Guangxi, 541004

b. Collaborative Innovation Center for Water Pollution Control and Water Safety in Karst Area, Guilin, Guangxi, 541004

c. School of Biomedical Engineering, Southern Medical University, Guangzhou, Guangdong, 510515

*Corresponding author: College of Chemistry and Bioengineering, Guilin University of Technology, Guilin, Guangxi, 541004

E-mail: guihuaruan@hotmail.com (G. Ruan)

1 These authors contributed equally.

Supplementary Figures

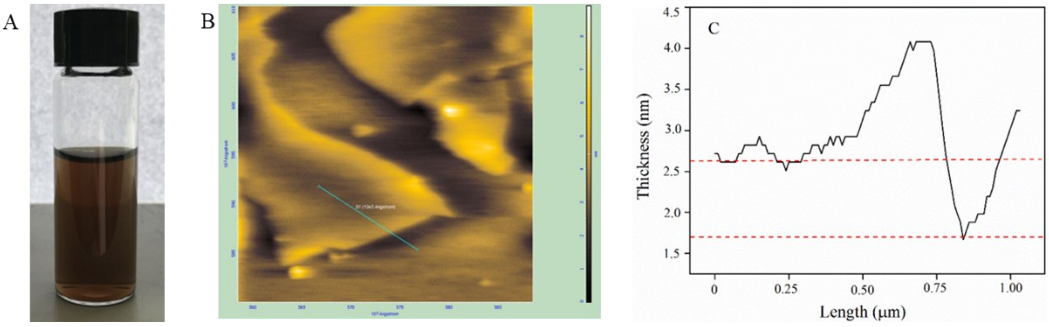

Fig. S1 Photograph of the PVP-GO aqueous suspension (A), and AFM image (B) and high profile (C) of the PVP-GO sheets.

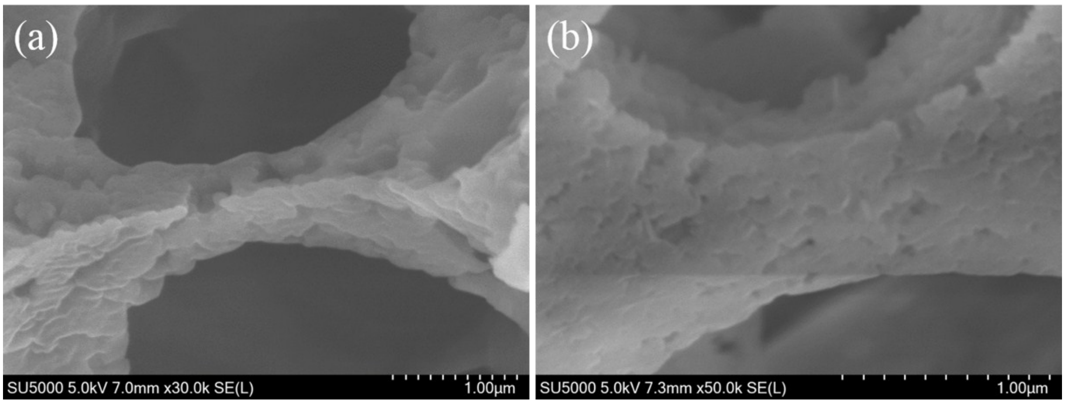

Fig. S2 SEM images for showing the nanoscale to submicroscale grooves on the pore walls in the as-prepared polyHIPEs/GO (a) and polyHIPEs$_{(NH2)}$/GO (b).

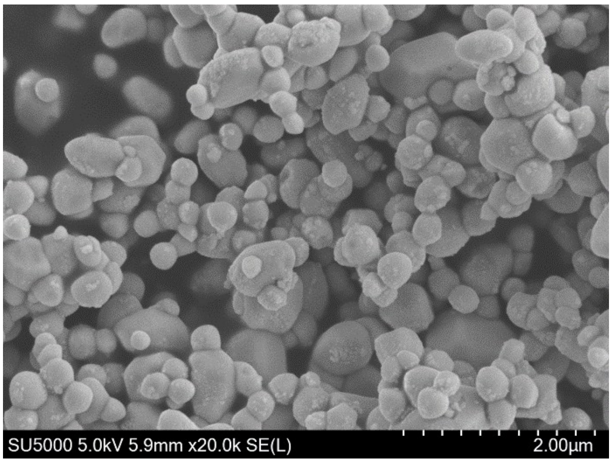

Fig. S3 The SEM image of the bared Ag_3PO_4 particles.

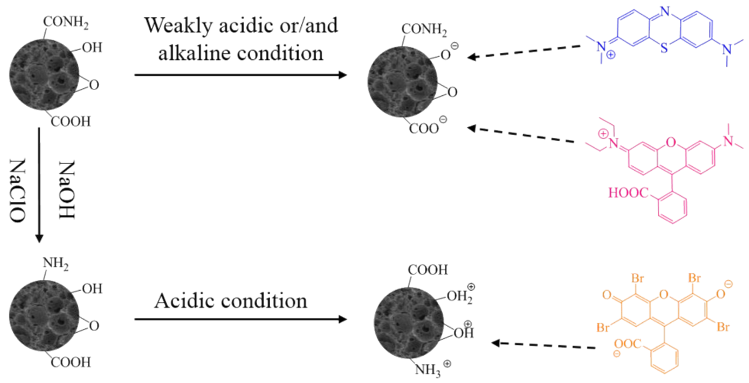

Fig. S4 Schematic illustration of the adsorption mechanism of dyes on polyHIPEs/GO and polyHIPEs$_{(NH2)}$/GO.

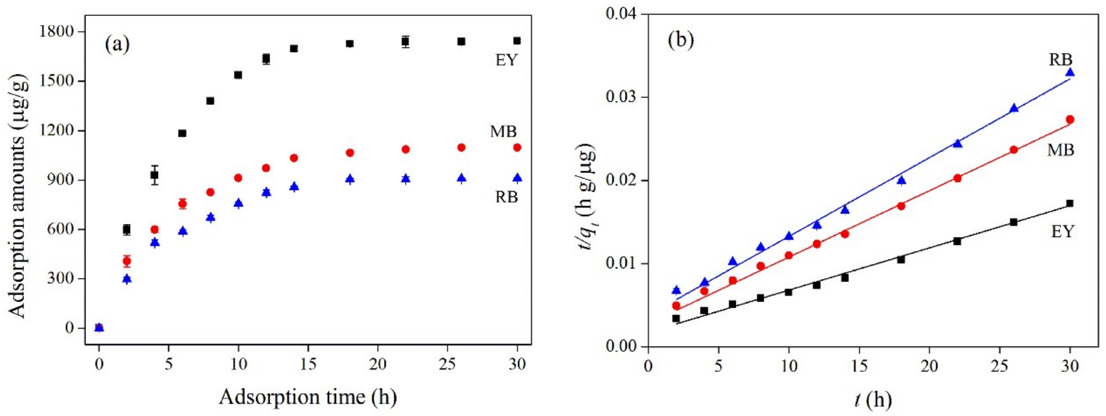

Fig. S5 (a) Adsorption amount of dyes versus contact time, (b) the pseudo-second-order kinetic curves of dyes.

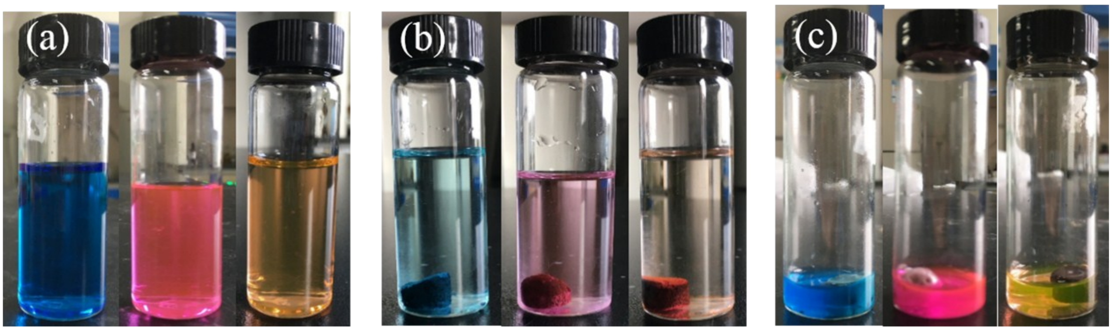

Fig. S6 Digital photographs of the MB, RB and EY solutions before adsorption (a), after adsorption for 14 h (b), and desorption in ethanol (c).

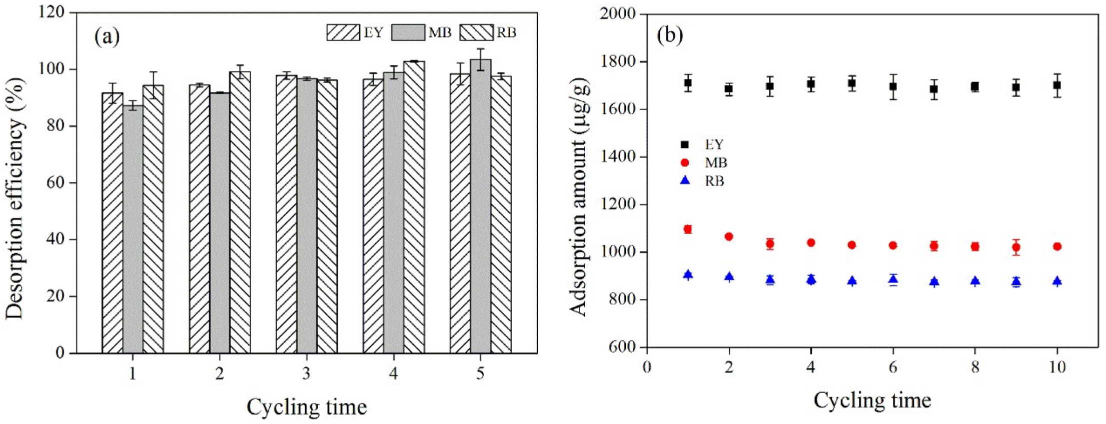

Fig. S7 The desorption efficiency of EY, MB and RB for the first 5 cycling time (a), and the cyclic performance of polyHIPEs/GO to MB and RB adsorption, and polyHIPEs$_{(NH2)}$/GO to EY (b).

Supplementary Tables

Tab. S1 The quantity used of AAm, DVB and PVP-GO in HIPE preparation

Sample	AAm (mg)	DVB (μL)	PVP-GO (mg)
1	0	300	10
2	50	300	10
3	100	300	10
4	100	0	10
5	100	100	10
6	100	300	10
7	100	300	0
8	100	300	5
9	100	300	10
10	100	300	15

Notes: To maintain the total volume of oil phase unchanged, the dosage of EHA is 700, 600, and 400 μL for sample 4, 5, and 6, respectively. Other reagents for other samples are the same as described in 2.3 in the text.

Tab. S2 Kinetics parameters for the adsorption of MB, RB using polyHIPEs/GO, and the adsorption of EY using polyHIPEs$_{(NH2)}$/GO

Dye	Pseudo-second-order kinetic equation	R^2	q_e (μg/g)	k (g μg^{-1} h^{-1})
MB	$t/q_t = 7.9977 \times 10^{-4}\, t + 0.0028$	0.9949	1250.3	2.30×10^{-4}
RB	$t/q_t = 9.4871 \times 10^{-4}\, t + 0.0038$	0.9990	1054.1	2.38×10^{-4}
EY	$t/q_t = 5.0830 \times 10^{-4}\, t + 0.0017$	0.9944	1967.3	1.49×10^{-4}

Tab. S3 Comparison of the dye adsorption performances of polyHIPEs/GO and polyHIPEs$_{(NH2)}$/ GO with other reported sorbents.

Adsorbents	Dyes	Adsorption capacity	Advantages	Existing issues	Ref.
Poly(1-vinylimidazole)/$_{88\%}$graphene	MB	1910 mg/g	High adsorption capacity	The synthesis of the sorbent is time consuming (it takes about one week in a typical procedure); the sorbent is high-cost; the 2D sheets are inconvenient to recycle from solutions	1
Silicon/carbon/nitrogen hybrids	MB<br>Acid fuchsin	1327.7 mg/g<br>1084.5 mg/g	High adsorption capacity	The adsorption only feasible to triphenyl dyes; the powder-like sorbent is inconvenient to recycle from solutions	2
Polyethylenimine/$_{33\%}$GO	Amaranth<br>Orange G<br>RB	800 mg/g<br>300 mg/g<br>25 mg/g	The adsorption capacity to acidic dyes is high; the 3D sorbent is very convenient to recycle from solutions	The adsorption to basic dyes is low; the sorbent is high-cost and easy to collapse	3
Chitosan/$_{91\%}$GO hydrogel	MB<br>EY	350 mg/g<br>230 mg/g	The sorbent shows broad-spectrum adsorption ability to both cationic and anionic dyes	The very high GO content make this sorbent costly; the hydrogel based sorbent needs to preserve in water; the cycling performance is questioned	4
Poly(vinylbenzyl chloride-divinylbenzene)/$_{90\%}$chitosan hydrogel	Indigo Carmine<br>Sunset Yellow<br>Rhodamine 6G	118 mg/g<br>72 mg/g<br>78 mg/g	The sorbent shows broad-spectrum adsorption ability to both cationic and anionic dyes	The synthesis procedure is time consuming; the hydrogel based sorbent needs to preserve in water	5
PolyHIPEs/$_{1.35\%}$GO, polyHIPEs$_{(NH2)}$/$_{1.35\%}$GO	MB<br>RB<br>EY	1250.3 µg/g<br>1054.1 µg/g<br>1967.3 µg/g	The sorbent shows broad-spectrum adsorption ability to both cationic and anionic dyes; the synthesis procedure is simple; the monolithic sorbent is cost-effective and ease of recycling	The adsorption capacity is relatively low	This work

Tab. S4 Comparison of the photocatalayatic activity of polyHIPEs$_{(NH2)}$/RGO/Ag_3PO_4 with some other graphene/semiconductor composites

Photocatalyst (dosage in mg)	Dye (dosage in mol)	Irradiation source	Degradation percentages	Time (min)	Catalytic efficiency (mol mg^{-1} min^{-1})	Ref.
RGO/$_{95\%}BiVO_4$ (100)	MB and RB (2.7×10^{-6})	300 W Xe, >400 nm	94% for MB. 87% for RB	30	8.5×10^{-10} MB, 7.8×10^{-10} RB	6
RGO/$_{70\%}$CdS (20)	RB (5.0×10^{-7})	500 W Xe	95%	80	3.0×10^{-10}	7
RGO/$_{90\%}TiO_2$ nanotube (20)	Malachite green oxalate (2.8×10^{-6})	450 W Hg	80%	75	1.5×10^{-9}	8
RGO/$_{99\%}TiO_2$ P25 (30)	MB (1.1×10^{-6})	100 W Hg, > 400 nm	42%	10	1.5×10^{-9}	9
RGO/$_{94.4\%}Ag_3PO_4$ (50)	MB, RB and methyl orange (1.0×10^{-6})	350 W Xe, >420 nm	Nearly 100%	5	4.0×10^{-9}	10
GO/$_{98.2\%}Ag_3PO_4$ (20)	Acid Orange (7.1×10^{-9})	300 W Xe, $420 < \lambda < 630$ nm	Nearly 100%	10	3.5×10^{-11}	11
GO/$_{92\%}Ag_3PO_4$ (35)	RB (1.7×10^{-6})	500 W Xe, >420 nm	Nearly 100%	22	2.2×10^{-10}	12
PolyHIPEs$_{(NH2)}$/$_{4.6\%}$RGO/$_{49.7\%}Ag_3PO_4$ (20)	MB, RB and EY (7.0×10^{-7})	350 W Xe, >420 nm	Nearly 100%	MB: 20, RB: 40, EY: 35	1.8×10^{-9} MB, 8.8×10^{-10} RB, 1.0×10^{-9} EY	This work

References

1 W. Zhao, Y. Tang, J. Xi and J. Kong, *Applied Surface Science*, 2015, **326**, 276-284.

2 L. Meng, X. Zhang, Y. Tang, K. Su and J. Kong, *Scientific Reports*, 2015, **5**, 7910.

3 Z.-Y. Sui, Y. Cui, J.-H. Zhu and B.-H. Han, *ACS Applied Materials & Interfaces*, 2013, **5**, 9172-9179.

4 Y. Chen, L. Chen, H. Bai and L. Li, *Journal of Materials Chemistry A*, 2013, **1**, 1992-2001.

5 d. L. M. Salzano, R. Castaldo, R. Altobelli, L. Gioiella, G. Filippone, G. Gentile and V. Ambrogi, *Carbohydrate Polymers*, 2017, **177**, 347-354.

6 Y. Wang, W. Wang, H. Mao, Y. Lu, J. Lu, J. Huang, Z. Ye and B. Lu, *ACS applied materials & interfaces*, 2014, **6**, 12698-12706.

7 Z. Gao, N. Liu, D. Wu, W. Tao, F. Xu and K. Jiang, *Applied Surface Science*, 2012, **258**, 2473-2478.

8 S. D. Perera, R. G. Mariano, K. Vu, N. Nour, O. Seitz, Y. Chabal and K. J. Balkus, *ACS Catalysis*, 2012, **2**, 949-956.

9 H. Zhang, X. Lv, Y. Li, Y. Wang and J. Li, *ACS Nano*, 2010, **4**, 380-386.

10 X. Yang, H. Cui, Y. Li, J. Qin, R. Zhang and H. Tang, *ACS Catalysis*, 2013, **3**, 363-369.

11 L. Liu, J. Liu and D. D. Sun, *Catalysis Science & Technology*, 2012, **2**, 2525-2532.

12 Q. Liang, Y. Shi, W. Ma, Z. Li and X. Yang, *Physical Chemistry Chemical Physics*, 2012, **14**, 15657-15665.
